# Correction to: ‘Conservation and turnover of miRNAs and their highly complementary targets in early branching animals’ (2021), by Praher *et al.*

**DOI:** 10.1098/rspb.2025.2588

**Published:** 2025-11-26

**Authors:** Daniela Praher, Yehu Moran, Ulrich Technau

**Affiliations:** ^1^ Department of Molecular Evolution and Development, University of Vienna, Vienna 1090, Austria; ^2^ Department of Ecology, Evolution and Behavior, Hebrew University of Jerusalem, Edmond J. Safra Campus, Jerusalem, Israel; ^3^ Department of Neurosciences and Developmental Biology, Faculty of Life Sciences, University of Vienna, Vienna 1030, Austria

**Correction note:**

The authors have recently become aware of errors in figures 3 and 4 of our paper by Praher *et al*. (2021) [1] titled ‘Conservation and turnover of miRNAs and their highly complementary targets in early branching animals’ published in *Proceedings of the Royal Society B* 288: 20203169. https://doi.org/10.1098/rspb.2020.3169

**Figure 3.**
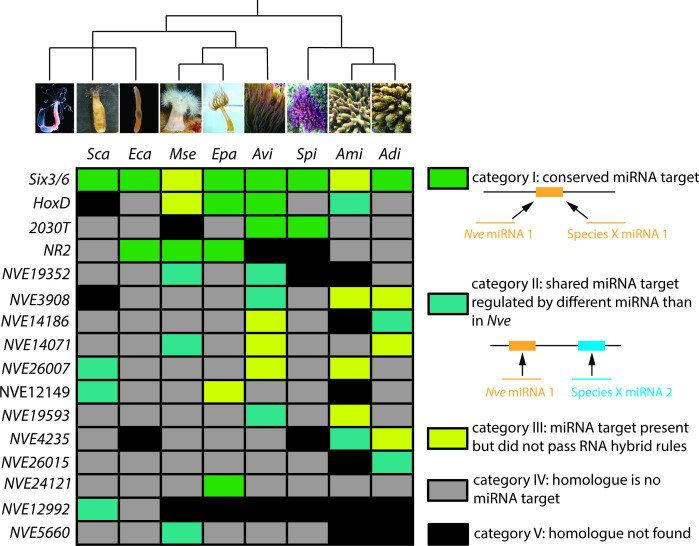
The colour of *HoxD* in *A. millepora* in the original version was wrong and hence was changed in the corrected version to the green used for ‘category II: shared microRNA (miRNA) target regulated by a different miRNA than in *N. vectensis*.’

**Figure 4A.**
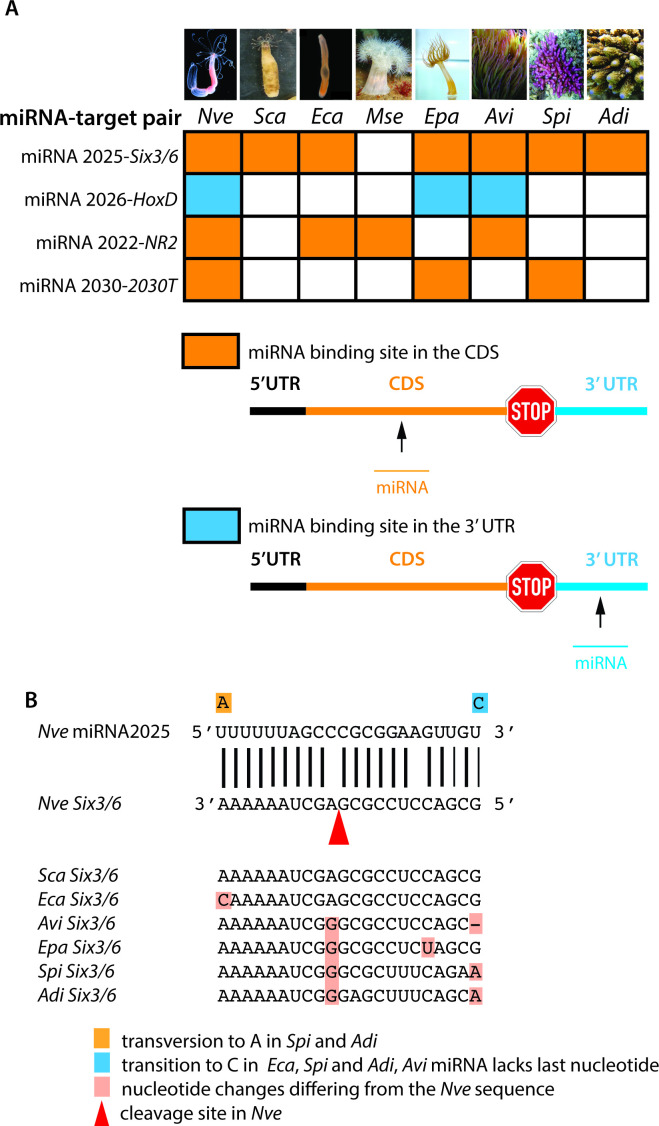
The column for *A. millepora* should not be present and was deleted in the corrected version. *HoxD* is a conserved target in *A. millepora* but is targeted by a different miRNA. Hence, the species name *A. millepora* was also removed from the corresponding figure legend. Furthermore, as miR-2026 does not exist in the coral *S. pistillata,* the colour of the box representing ‘miRNA 2026-HoxD’ should not be blue, but white, as was corrected in the new version.

In brief, miR-2026 does not exist in *Acropora millepora* or any other reef-building coral (as correctly shown in figure 1), and therefore the following corrections are required.

**Main text**

The sentence ‘The number of conserved miRNA targets ranges between three and seven with *Six3/6* being the most conserved one in all anthozoan species sequenced (figures 3 and 4)’ was corrected to ‘The number of conserved miRNA targets ranges between three and seven with *Six3/6* and *NR2* being the most conserved one in all anthozoan species sequenced (figures 3 and 4)’.
